# Analysis of Narrative Text in Evaluations of Continuing Medical Education Faculty by Gender

**DOI:** 10.1001/jamanetworkopen.2022.27948

**Published:** 2022-08-22

**Authors:** Lekshmi Santhosh, Alexandra E. Rojek, Joanne W. L. Yim, Sarah Lisker, Katarina Wang, Marika Dy, Urmimala Sarkar

**Affiliations:** 1 Department of Medicine, University of California, San Francisco; 2 Department of Medicine, University of Chicago, Chicago, Illinois; 3 Health Informatics, UCSF Health, University of California, San Francisco; 4 UCSF Center for Vulnerable Populations, San Francisco, San Francisco

## Abstract

This mixed-methods study examines the performance ratings and feedback received by female and male lecturers at national internal medicine conferences.

## Introduction

Free-text comments in evaluations are important adjuncts to quantitative assessments yet may present opportunities for implicit biases, particularly for those underrepresented in their fields. Analysis of medical school clerkship evaluations found substantial gender- and race-based differences in narrative evaluations,^1^ and other studies reported use of gendered and racialized adjectives in summative evaluations of medical students and other medical trainees.^2,3,4^

One study of faculty evaluations by trainees found no gender-based differences, but comments tended to focus on female faculty demeanor.^5^ To our knowledge, no research has been conducted on gender bias in continuing medical education (CME) faculty evaluations. In this study, we explored whether CME evaluations describe faculty differently by gender.

## Methods

We analyzed evaluations of University of California, San Francisco–sponsored national CME conferences on internal medicine and its subspecialties between 2015 and 2019, and we pulled speakers’ gender information (male or female) from university websites. Evaluations included a numeric rating scale (range: 1-5, with 5 indicating the most favorable) for syllabus content, practical application, and presentation delivery, with free-text qualitative comments. We applied natural language processing techniques to evaluation data using the same methods used in Rojek et al.^1^ We also manually coded comments as advice-giving or not. All analyses were performed in R, version 4.0.2 (R Foundation for Statistical Computing), and 2-sided *P* < .05 was considered to be significant. The University of California, San Francisco Institutional Review Board approved this mixed-methods study and waived the informed consent requirement because deidentified data were used. We followed the relevant parts of the SRQR reporting guideline as well as the STROBE reporting guideline.

## Results

We analyzed 38 026 evaluations of 2149 CME lectures. A total of 21 557 evaluations (56.7%) were for male faculty and 16 469 evaluations (43.3%) were for female faculty (Table 1). Female speakers compared with male speakers scored higher in syllabus content (3.96 vs 3.95; *P* = .02) and practical application (3.98 vs 3.96; *P* = .04), with no significant differences in presentation delivery score. Evaluations for 60% of female speakers and 55% of male speakers included advice-giving comments (*P* = .03).

**Table 1.** Descriptive Characteristics and Quantitative Evaluation Scores by Faculty Gender

	Female faculty, No. (%)	Male faculty, No. (%)	Total No.
No. of sessions evaluated	784 (42.5)	1060 (57.5)	1844
Total No. of evaluations	16 469 (43.3)	21 557 (56.7)	38 026
Specialty			
Asian health	22 (57.9)	16 (42.1)	38
Cardiology	35 (16.7)	175 (83.3)	210
Critical care	7 (3.3)	19 (9.0)	26
Emergency medicine	1 (8.3)	11 (91.7)	12
Endocrinology	20 (55.6)	33 (91.7)	36
Gastroenterology	6 (11.3)	19 (35.8)	53
Infectious disease	226 (59.8)	152 (40.2)	378
Inpatient care	8 (25.0)	24 (75.0)	32
Internal medicine	280 (41.5)	394 (58.5)	674
Primary care	85 (46.2)	99 (53.8)	184
Pulmonary medicine	4 (21.1)	15 (78.9)	19
Rheumatology	90 (46.6)	103 (53.4)	193
Length of evaluations (No. of words), mean (SD)	18 (SD)	18.3 (SD)	NA
Score^a^			
Delivery	3.98	3.97	NA
Content	3.99	3.99	NA
Syllabus^b^	3.96	3.95	NA
Practical^c^	3.98	3.96	NA
Average	3.98	3.97	NA

Abbreviation: NA, not applicable.

^a^ Range 1-5, with 5 representing the most favorable.

^b^ *P* = .02.

^c^ *P* = .04.

The most used adjectives overall and within gender groups were similar. Female faculty evaluations were more likely to contain *important, knowledgeable, packed, essential,* and *information*. Male faculty evaluations were more likely to contain *entertaining, interesting, fun, enjoyed*, and *dynamic*. Although some adjectives appeared with similar frequency across genders, contextual use of some words may have negative connotations (Table 2).

**Table 2.** Contextual Use of Words in Comments

Word	Evaluations	
	Female faculty	Male faculty
**Terms used substantially more in female faculty evaluations**		
Knowledgeable	“Very knowledgeable and a good speaker, but I was losing interest, especially later in the day.”	“What an entertaining and knowledgeable professor!”
	“You are a great, dynamic speaker, just stop the ‘ums’ and ‘uhs.’ Very knowledgeable.”	“Clearly very knowledgeable about his topic, but delivery was not engaging.”
	“Thankful she is compassionate, knowledgeable and motivated!”	“Extremely knowledgeable and comfortable.”
	“Very knowledgeable, but hard speaker to follow, fast and monotonous.”	“Speaker is knowledgeable, and very practical.”
	“She is a little flat in delivery, but knowledgeable.”	“He is knowledgeable, needs to incorporate questions.”
Important	“Important topic to understand. Interesting to have a panel with different views.”	“The content is important, but I’m not sure if it’s communicated well.”
	“Very important topic, good resources, thank you!”	“Very practical, full of important updates and paradigm changing.”
	“Important topic. Her voice faded at times.”	“Important topic, but difficult to follow.”
	“A bit too much content, but good info. Important however.”	“Slow down—it is more important to get major points across than to cover 10+ different subjects.”
	“Well organized, important info, practical and nicely tied in to the ‘big picture.’”	“Great slow speaking, emphasis of important points and repetition, very helpful info.”
Essential	“Absolutely essential speaker.”	“Essential message, solidarity.”
	“Would recommend paring down the content of what is most essential to more efficiently promote learning/retention.”	“I’ve heard other people speak on this, but you convinced me that I can do this and that it is essential to good care.”
	“A major disappointment for an essential subject, who assigned this poor lady this talk?”	“This talk was very informative and provided essential updates I will use in my everyday practice.”
	“Too many ‘uhs,’ you put me to sleep with your delivery. This content is essential and can be delivered better.”	
**Terms used substantially more in male faculty evaluations**		
Entertaining	“Exceptional keynote speaker! Great end of the day speaker…very entertaining, great delivery of the tough topic.”	“Best speaker so far! Entertaining, clear and very practical.”
	“As usual, very knowledgeable and entertaining.”	“Always entertaining and super practical. Best speaker of CME.”
	“Engaging, entertaining, yet educational.”	“Entertaining, practical, clinically based! Great!”
	“Entertaining and lively speaker, which was good at the end of the day. I liked that she wrapped in her own story.”	“Exceptional speaker. Entertaining and memorable.”
	“Entertaining talk! Her style makes it easier to pay attention after lunch.”	“Outstanding, effective, entertaining teacher. Please teach other physician’s how to teach, including me!”
Interesting	“Great talk, interesting. Well organized, useful points.”	“Most interesting part of conference, keep it going.”
	“Could have been more interesting.”	“Last but the BEST of all, so interesting, keep coming!”
	“Overall interesting but not the most high yield in terms of my clinical practice.”	“Uncommon cases, but still very interesting and entertaining.”
	“Interesting discussion of common and uncommon presentations.”	“Interesting case. So valuable to watch a master clinical mind at work.”
	“Audience participation makes the talk more lively and interesting instead of looking at your own slides throughout the presentation.”	“Interesting intellectual exercise.”
Dynamic	“Outstanding talk, great slides, dynamic speaker.”	“Excellent, very dynamic, high yield.”
	“Dynamic talk, very helpful, practical! Data-based!”	“Fantastic, good content, photos, dynamic speaker, also practical.”
	“Not a dynamic speaker, could much improve with coaching.”	“Dynamic speaker, lots of info, helpful and quite informative.”
	“Need a more dynamic delivery. Pace was too slow.”	“Best delivery this speaker has given, more dynamic presentation vs. others given.”
	“Interesting preventative info, dynamic presentation.”	“Very useful lecture with a dynamic speaker.”

## Discussion

We found no significant differences by gender in quantitative ratings and frequency of adjectives. It is possible that increased awareness of implicit bias in assessment plays a role in evaluators’ more conscious and less gendered use of language as more studies examine and discuss implicit bias. One could hypothesize that faculty members have less implicit bias when assessing each other than when evaluating trainees, with whom they have long-term, individual interactions. However, we noticed subjective differences when examining contextual use of some adjectives, some of which may be associated with unfavorable contextual sentiments, which remains difficult to quantify. When comparing frequencies of evaluations that included advice-giving, we found that female speakers were significantly more likely than male counterparts to receive advice, although further work is needed to clarify whether such comments were constructive feedback or biased criticism.

Female speakers may also receive a likeability penalty when perceived as competent at a task.^6^ They may modify their presentation delivery style to balance on the competency-likeability axis, erring on the side of being more knowledgeable rather than too dynamic. Although there were no specific differences in the language used to evaluate male and female speakers, subtle differences may be highly contextual and associated with implicit gender bias.

Study limitations include analysis of only anonymous evaluations from internal medicine conferences and unavailability of race and ethnicity data. We also did not distinguish between favorable and unfavorable word context. Speakers received high numerical scores overall, but a greater spread of ratings may have shown other findings.
